# Supplementary material for: Mef2c- and Nkx2-5-Divergent Transcriptional Regulation of Chick WT1_76127 and Mouse Gm14014 lncRNAs and Their Implication in Epicardial Cell Migration
Source: Int J Mol Sci. 2024 Nov 30;25(23):12904. doi: 10.3390/ijms252312904 (PMC11640978; doi:10.3390/ijms252312904)
Supplement: Supplementary file 1 [file ijms-25-12904-s001.zip › Supplementary Figures and Tables legends.pdf]

Supplementary Figures and Tables legends

Supplementary Figure S1. Panel A. RT-qPCR validation of *Mef2c, Nkx2.5*, *Pitx2c* and *Srf* siRNA inhibition in epicardial/ventricular samples, respectively. Panel B,C. RT-qPCR analyses of *Wt1_76127*, *Bmp4_53170* and *Fgf8_57126* nuclear and cytoplasmic expression in controls and *Mef2c* siRNAs treated of HH24 and HH32 epicardial/ventricular tissues, respectively. Note that inhibition of *Mef2c* selectively modulates nuclear and cytoplasmic expression in different tissues and stages.

Supplementary Figure S2. Panel A. Graphical representation of the secondary structure of *Wt1_76127* lncRNA. Panel B. Graphical representation of the secondary structure of *Gm14014* lncRNA. Red areas in *Gm14014* correspond to the conserved nucleotide sequences in *Wt1_76127*. The colour of nucleotides represents the probability of base pairs.

Supplementary Figure S3. Panel A. RT-qPCR validation of *Xist2* expression as a nuclear marker in nuclear and cytoplasmic extracts of HL1 cardiomyocytes, MEC1 and EPIC epicardial cells and MEVEC endocardial cells, respectively. Panel B. RT-qPCR validation of *Mef2c*, *Nkx2.5*, *Pitx2c* and *Srf* siRNA inhibition and overexpression in HL1 cardiomyocytes, MEC1 and EPIC epicardial cells and MEVEC endocardial cells, respectively. Panel C. Global view of SCRINSHOT in situ hybridization analyses of *Gm14014* in E14.5 mouse hearts. Global view of SCRINSHOT in situ hybridization analyses of *Gm14014* in P21 ventricular (1) and atrial (2) close-ups. Panel D. Quantitative analysis of SCRINSHOT *in situ* hybridization for *Gm14014* expression in E14.5 and P21 samples. Note that *Gm14014* expression is higher in embryonic stage than P21.

Supplementary Figure S4. Panel A. Schematic representation of the gene ontology analyses corresponding to the biological processes of the *Gm14014* interacting proteins as revealed by RNA pull-down assays and mass spectrometry identification. Panel B. Schematic representation of the gene ontology analyses corresponding to cellular components of the *Gm14014* interacting proteins as revealed by RNA pull-down assays and mass spectrometry identification. Panel C. Schematic representation of the gene ontology analyses corresponding to the molecular functions of the *Gm14014* interacting proteins as revealed by RNA pull-down assays and mass spectrometry identification.

Supplementary Figure S5. Panel A. RT-qPCR validation analyses of *Gm14014* after ASO administration, corresponding to ASO1, ASO2 and ASO1+ASO2 conditions, respectively. Panel B. Schematic representation of the direction of epicardial MEC1 cells in the migration assay between control condition and *Gm14014* ASO treatment. These data represent the graph in **Figure 4** (panel 4D). Panel C. RT-qPCR validation analysis of *Myl9* after siRNA and ASO of *Myl9* administration. Panel D and E. Representative immunocytochemical images of MYH9 and MYL9 expression corresponding to HL1 cardiomyocytes after 6h (panel D) and 24h (panel E) ASO1 administration, respectively and their corresponding quantitative analyses graphs. Observe that no significant differences are observed at any of the two distinct timepoints analyzed.

Supplementary Figure S6. Panel A. Schematic representation *in ovo* of *Wt1_76127* ASO pericardial administration in HH17 chicken embryos. Panel B. Graphical representation of the survival rate in controls and HH17 *Wt1_76127* ASO treated embryos. Panel C. Graphical representation of the beats per minute frequency in controls and HH17 *Wt1_76127* ASO treated embryos. Panel D. Graphical representation of cardiac rhythm rhythmicity in controls and HH17 *Wt1_76127* ASO treated embryos. Panel E. Graphical representation of heart surface in controls and HH17 *Wt1_76127* ASO treated embryos. Observe that none of the parameters analyzed is significantly different between controls and HH17 *Wt1_76127* ASO treated embryos.

Supplementary Table S1. List of the nucleotide sequences corresponding to RT-qPCR primers, biotinylated RNAS primers for RNA pulldown assays, siRNAs, antisense oligonucleotides (ASOs) SCRINSHOT padlock probes and SCRINSHOT detection oligonucleotides.

Supplementary Table S2. Comparative analyses of nucleotide conservation between the chicken *Wt1_76127* lncRNA and the mouse *Gm14014* lncRNA.

Supplementary Table S3. List of *Gm14014* associated proteins as revealed by RNA pulldown assays and mass spectrometry identification.
