# Supplementary material for: Mef2c- and Nkx2-5-Divergent Transcriptional Regulation of Chick WT1_76127 and Mouse Gm14014 lncRNAs and Their Implication in Epicardial Cell Migration
Source: Int J Mol Sci. 2024 Nov 30;25(23):12904. doi: 10.3390/ijms252312904 (PMC11640978; doi:10.3390/ijms252312904)
Supplement: Supplementary file 1 [file ijms-25-12904-s001.zip › Supp_Figures.pdf]

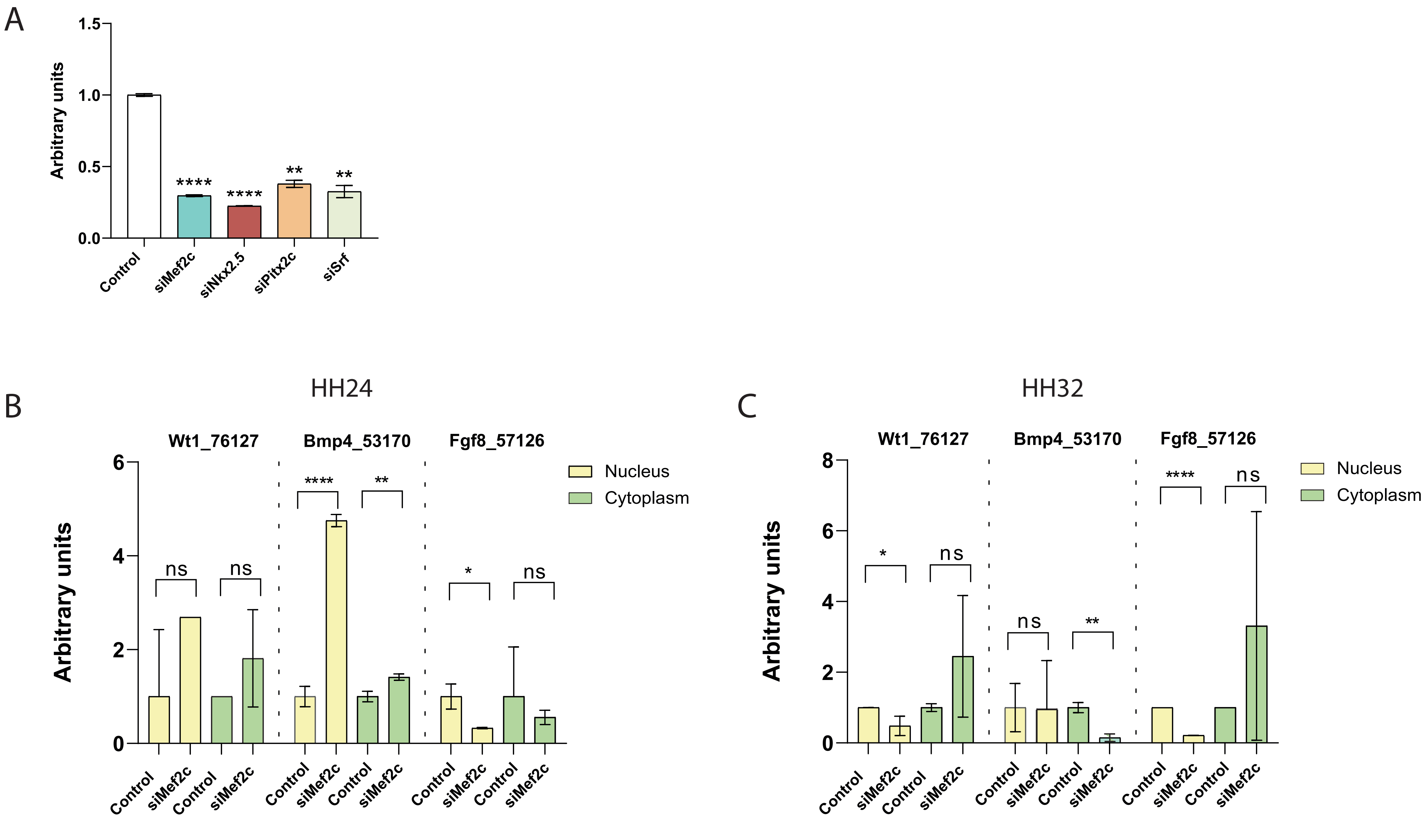

Supplementary Figure S1

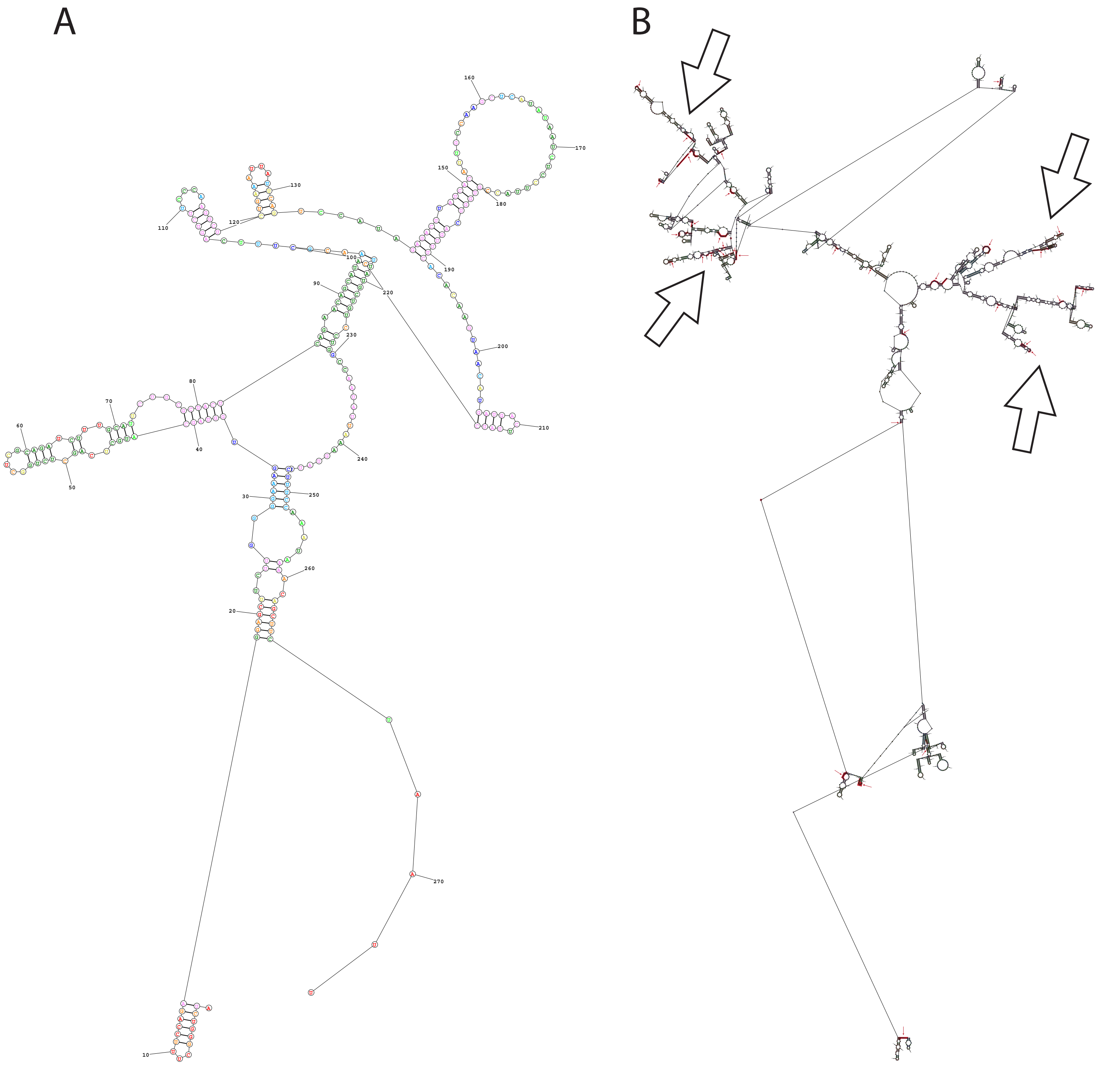

Energy = -66.5   Wt1_76127

Energy = -728.8 Gm14014

Probability >= 99%
99% > Probability >= 95%
95% > Probability >= 90%
90% > Probability >= 80%
80% > Probability >= 70%
70% > Probability >= 60%
60% > Probability >= 50%
50% > Probability

Supplementary Figure S2

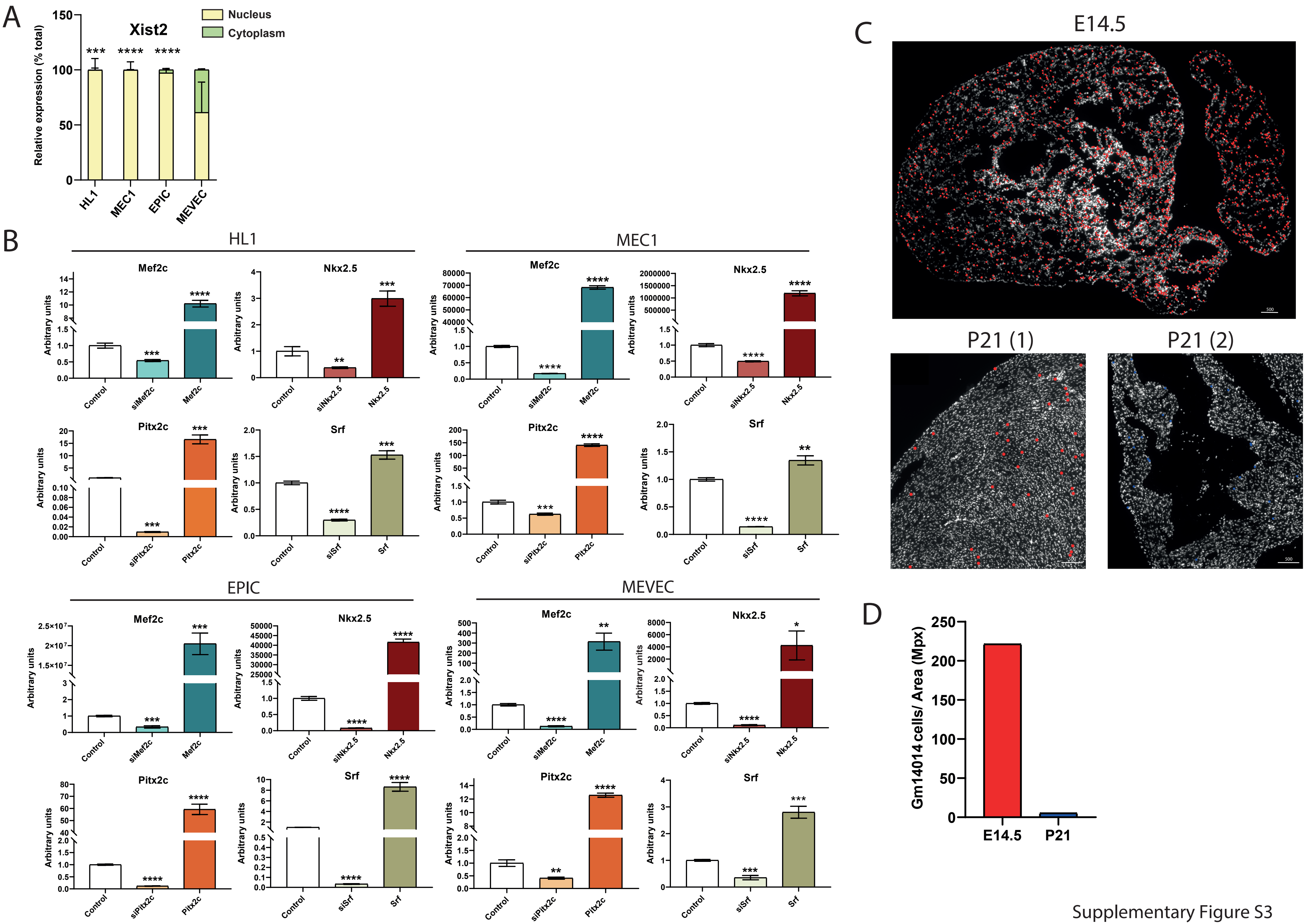

Supplementary Figure S3

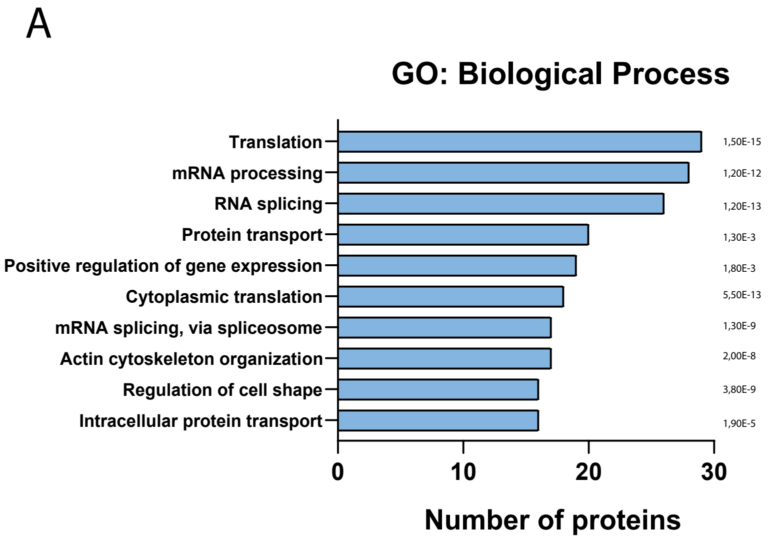

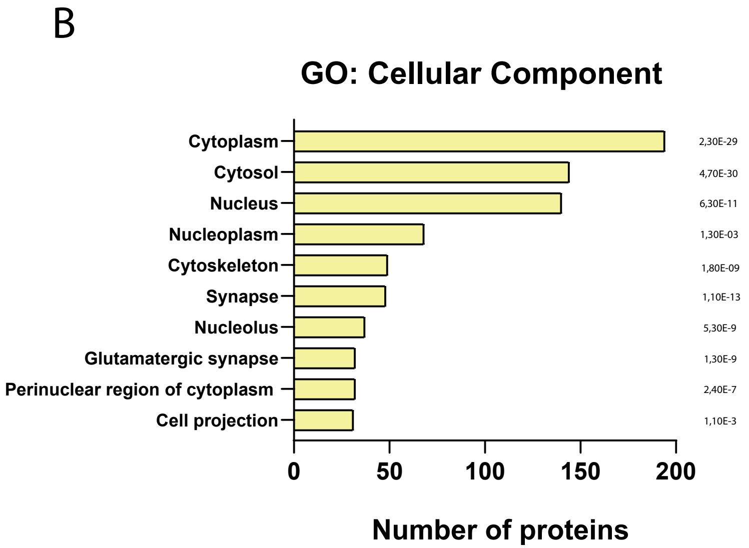

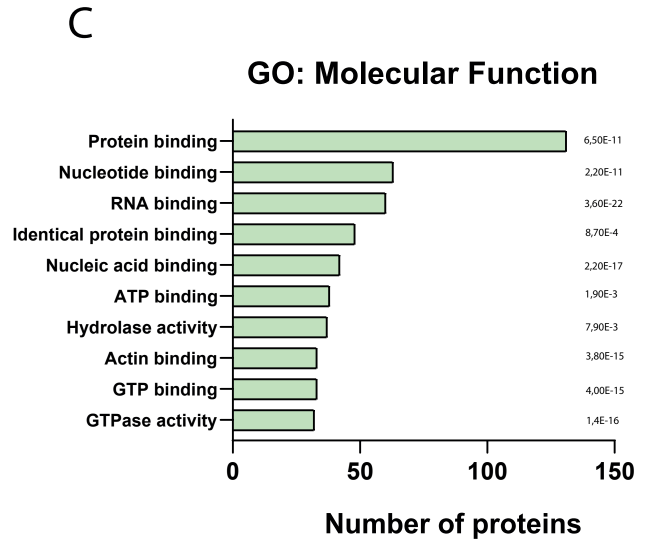

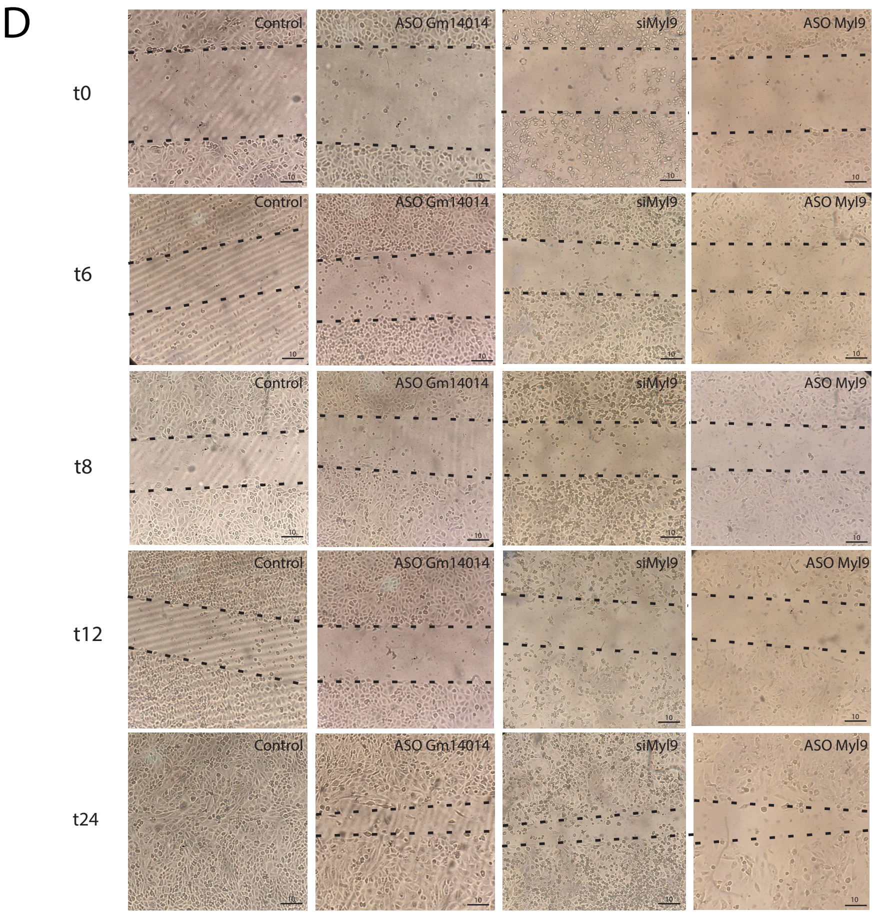

Supplementary Figure S4

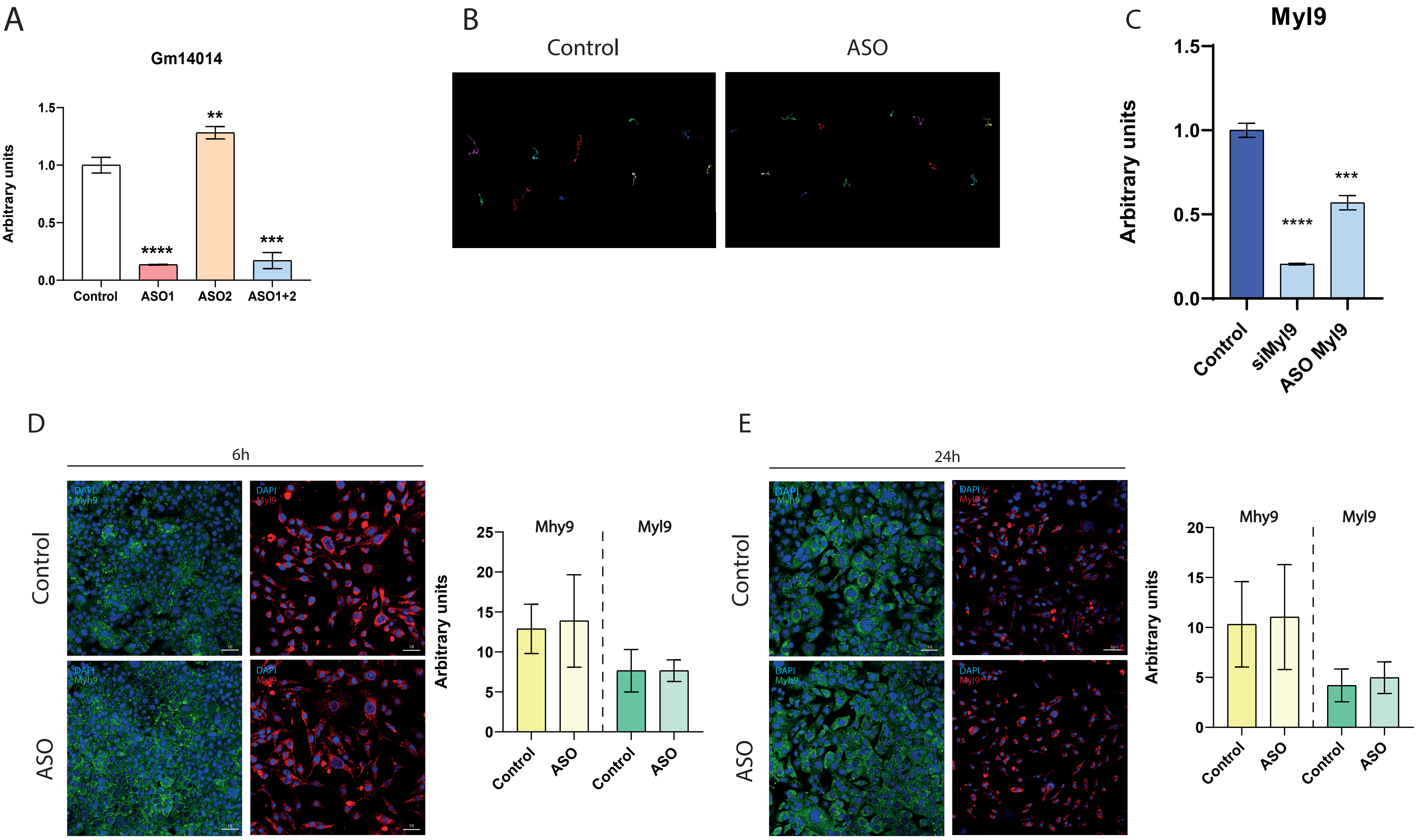

Supplementary Figure S5

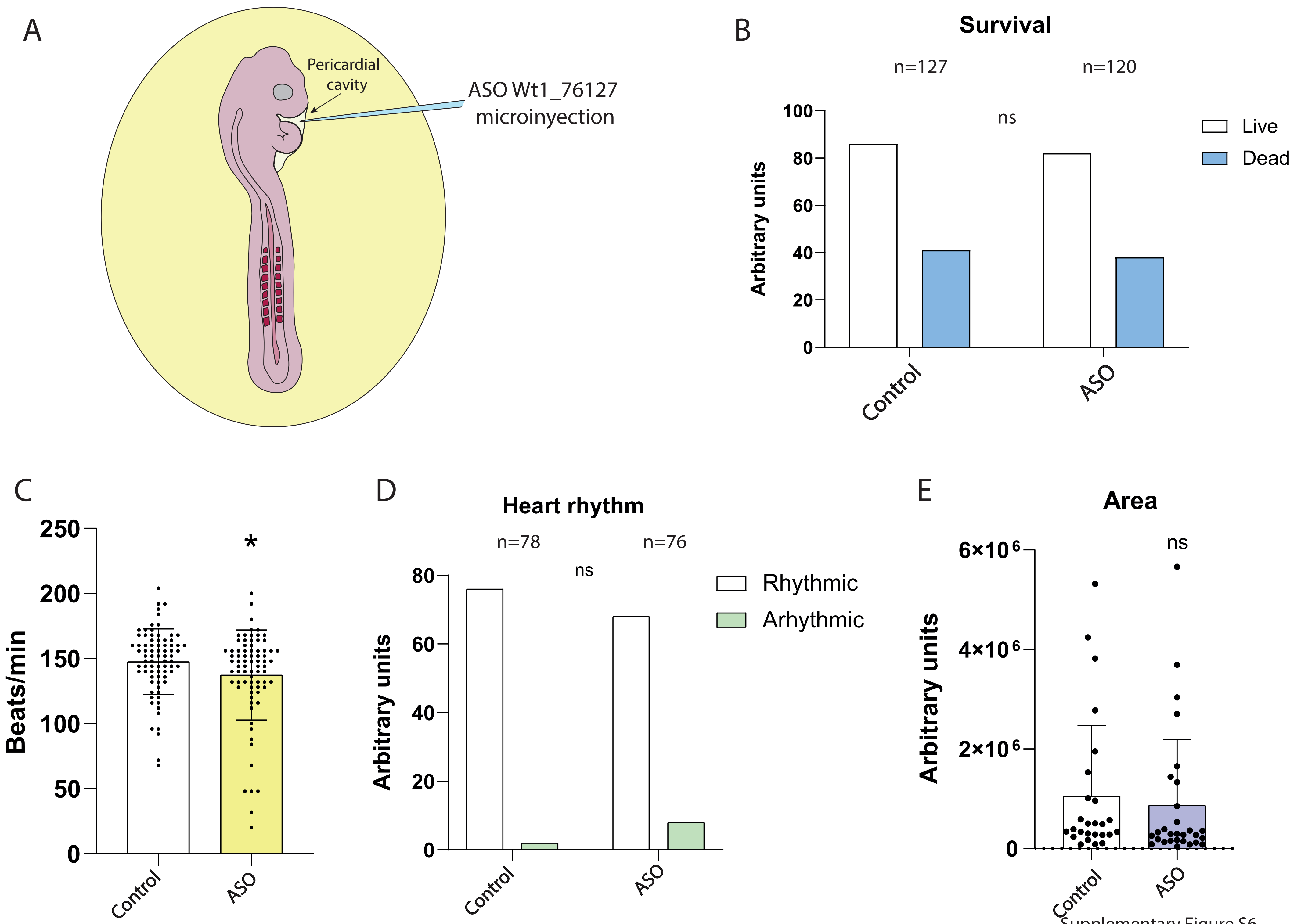

Supplementary Figure S6

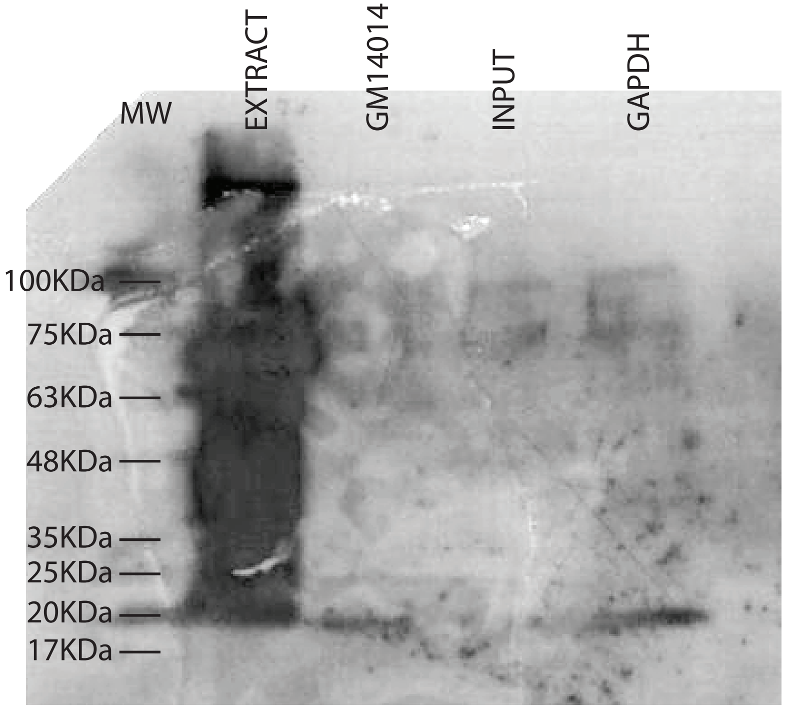

MW
EXTRACT
GM14014
INPUT
GAPDH
100KDa
75KDa
63KDa
48KDa
35KDa
25KDa
20KDa
17KDa

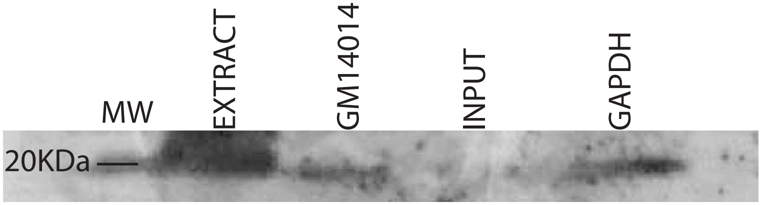

MW
EXTRACT
GM14014
INPUT
GAPDH
20KDa
