# Supplementary material for: Mef2c- and Nkx2-5-Divergent Transcriptional Regulation of Chick WT1_76127 and Mouse Gm14014 lncRNAs and Their Implication in Epicardial Cell Migration
Source: Int J Mol Sci. 2024 Nov 30;25(23):12904. doi: 10.3390/ijms252312904 (PMC11640978; doi:10.3390/ijms252312904)
Supplement: Supplementary file 1 [file ijms-25-12904-s001.zip › Supp_Tables.pdf]

Supplementary Table S1

Primers	
Gg_Wt1_76127	Fw: GGCCTGAGGGTGGTAGGG
	Rv: CCAAGAAGCTCCTCTGGAAA
Gg_Bmp4_53170	Fw: TCCGTGGCTGTAACTGTTTG
	Rv: TGCTGTAGGAGGCCAAGTCT
Gg_Fgf8_57126	Fw: CCAAGCAGGAGATGAGATCC
	Rv: ACGCATCTGTGTTTGACTGC
Gg_Mef2c	Fw: CCTCAGTCAACTGGGAGACC
	Rv: AGTGGAATTCATTCCGGTGA
Gg_Nkx2.5	Fw: TGAAGAGCTACGGGGAGATG
	Rv: TTGGGCTTGAGAAAAGAGGA
Gg_Pitx2c	Fw: AGGACCCCTCCAAGAAGAAG
	Rv: TCTTCCTCCATTTGGCTCTG
Gg_Srf	Fw: CACCGTTCAAATCCCAGTCT
	Rv: TCCGTACGAGAGAGTTGCTG
Gg_Actn1	Fw: TCCAGGCCTTCATTGACTTC
	Rv: GGTCACTTTCGCCATAGAGC
Gg_Actn4	Fw: GGCCTTCATCGACTTCATGT
	Rv: GCGGTGGAGAAGGATTTGTA
Gg_Myh9	Fw: CCCTCAAGAGCAAACTCAGG
	Rv: TCATGTGTAACCTGGGCAAA
Gg_Gapdh	Fw: TGTCCTCTCTGGCAAAGTCC
	Rv: TGCCCATTGATCACAAGTTT
Mm_Gm14014	Fw: TGTCAAGCTGAGCTCCAAACT
	Rv: TCCTGAACCTTGCGGTACTG
Mm_Xist (isoform 2)	Fw: CCGTTCCTCACACCAGACTG
	Rv: AACAGCAAACTACCCCACCC
Mm_Mef2c	Fw: TGGTTTCCGTAGCAACTCCT
	Rv: AGTTACAGAGCCGAGGTGGA
Mm_Pitx2c	Fw: CCTCACCCTTCTGTCACCAT
	Rv: GCCCACATCCTCATTCTTTC
Mm_Srf	Fw: GCTCAATGCCTTCTCTCAGG
	Rv: CCCTATCACAGCCATCTGGT
Mm_Gata4	Fw: TCTCACTATGGGCACAGCAG
	Rv: CGAGCAGGAATTTGAAGAGG
Mm_Nkx2.5	Fw: TTGGCGTCGGGGACTTGAAC
	Rv: GGTGGGTGTGAAATCCGAGGGAC
Mm_Tnnt2	Fw: TTCGACCTGCAGGAAAAGTT
	Rv: GCACAGCTTTGACGAGAACA
Mm_Wt1	Fw: ACCATCTGAAGACCCACACC
	Rv: TCTGATGCATGTTGTGATGG
Mm_Tbx18	Fw: GGCCATCATTACGGACTCTC
	Rv: GGAGCAAGAGGATCCAGACA
Mm_Tcf21	Fw: CGCTCACTTAAGGCAGATCC
	Rv: TCACCACTTCCTTCAGGTCA
Mm_Snail1	Fw: CTTGTGTCTGCACGACCTGT
	Rv: AGTGGGAGCAGGAGAATGG
Mm_Snail2	Fw: CATTGCCTTGTGTCTGCAAG
	Rv: GATGTGCCCTCAGGTTTGAT
Mm_Cdh5	Fw: TGCATCCTCACCATCACAGT
	Rv: AGTGACCAACTGCTCGTGAA

Mm_Myh9	Fw: CACAGAGACCGCTGATGCTA<br>Rv: GACCTCCTCGTCTGAGCAGT
Mm_Myl9	Fw: ATCTGGAGGGCATGATGAAC<br>Rv: CATCTCGTCCACCTCCTCAT
Mm_Gapdh	Fw: GGCATTGCTCTCAATGACAA<br>Rv: TGTGAGGGAGATGCTCAGTG
Biotinylated RNA primers	
Mm_Gm14014_B1	Fw: AGTAATACGACTCACTATAGGGCTGCCTTCACAAGCTGAAAT<br>Rv: TTGCGGTACTGCCTTTCTTT
Mm_Gm14014_B2	Fw: AGTAATACGACTCACTATAGGGCGCAAGGTTCAGGAAAAGAG<br>Rv: ATTCTTCACCCCTCCTCCAT
Mm_Gm14014_B3	Fw: AGTAATACGACTCACTATAGGGGCAGCCACAGAATGTCTGAA<br>Rv: TCATGATTCCAGGGAGGAAG
Mm_Gm14014_B4	Fw: AGTAATACGACTCACTATAGGGCTCCCTGGAATCATGAGGAA<br>Rv: TGCTGCACAAAACAACAACA
Mm_Gm14014_B5	Fw: AGTAATACGACTCACTATAGGGTGGGAAACCTGGTGAGAAAC<br>Rv: TGGGGTGCTTATGAAGGAAG
Mm_Gm14014_B6	Fw: AGTAATACGACTCACTATAGGGTCCTTCATAAGCACCCCAAG<br>Rv: AAATTTACTGCCCCGTGTTG
Mm_Gm14014_B7	Fw: AGTAATACGACTCACTATAGGGCACGGGGCAGTAAATTTCAA<br>Rv: CGGGGGACAGAACTGTATTC
Gapdh_B	Fw: AGTAATACGACTCACTATAGGGCAGCAAGGACACTGAGCAAG<br>Rv: GGGTGCAGCGAACTTTATTG
siRNAs	
siPitx2c	S 5’ GUGCAUACAAUCUCCGAUAUU<br>AS 3' UUCACGUAUGUUAGAGGCUAU
siMef2c	S 5’ GGGUCGAUAUGCAUUAUAUUU<br>AS 3’ UUCCCAGCUAUACGUAAUAUA
siSrf	S 5' GACGGCACCACUUAUUUAUUU<br>AS 3’ UUCUGCCGUGGUGAAUAAAUA
siNkx2.5	S 5' CCAUUAAAGUGAGUGCGCUUU<br>AS 3’ UUGGUAAUUUCACUCACGCGA
siMyl9	S 5'GGCACUAACUAGAAGGGAU UU<br>AS 3' UUCCGUGAUUGAUCUUCCCUA
ASOs	
Wt1_76127 (ASO1)	52MOErT/*/i2MOErC/*/i2MOErA/*/i2MOErG/*/i2MOErG/*T*G*A*G*C*C*C*C*T*G*/i2MOErA/*/i2MOErG/*/i2MOErG/*/i2MOErG/*/32MOErC
Wt1_76127 (ASO2)	52MOErC/*/i2MOErC/*/i2MOErA/*/i2MOErT/*/i2MOErA/*G*A*G*A*C*T*T*A*C*A*/i2MOErC/*/i2MOErA/*/i2MOErT/*/i2MOErT/*/32MOErG
Gm14014 (ASO1)	mA*mA*mC*mU*mA*C*T*G*C*A*C*T*T*C*A*T*T*mG*mG*mU*mA*mC
Gm14014 (ASO2)	mG*mU*mC*mG*mU*G*T*C*C*A*C*A*G*T*T*T*T*mA*mC*mC*mG*mA
Myl9 ASO	mG*mG*mU*mA*mG*C*C*A*G*T*C*A*C*C*T*A*C*mG*mA*mG*mG*mG
SCRINSHOT padlock probes	
Mm_Acta2_pr1	TTCCTCTGTTGAAGTGATTGTCCTCTATGATTACTGACTGCGTCTATTTAGTGG<br>AGCCCCGTCTATCTTCTTTTTTACATCATTTAAGTGGACC
Mm_Acta2_pr2	AGCACAGCTTCTCCTTGATTCCTCTATGATTACTGACTGCGTCTATTTAGTGGA<br>GCCCCGTCTATCTTCTTTTTTCAAAGTCCAGAGCTACAT
Mm_Acta2_pr3	CCCATTCTGATAGGCAAAGTCCTCTATGATTACTGACTGCGTCTATTTAGTGGA<br>GCCCCGTCTATCTTCTTTCTCAAATAAACCTCTGTAGTT
Mm_Col1a2_pr1	GAGTTGCATCAACTTCATAGTCCTCTATGATTACTGACTGCGTCTATTTAGTGGAGC<br>CTATCCTATCTTCTTTATCTGGTTATTGAGAGATTTCA

Mm_Col1a2_pr2	TTAGCACCTTTGTGTTGCATCCTCTATGATTACTGACTGCGTCTATTTAGTGGAGCC TATCCTATCTTCTTTTCAAGTATCTGAAATACTACTAA
Mm_Col1a2_pr3	AATGCTGAATCTAATGAAGAGTCCTCTATGATTACTGACTGCGTCTATTTAGTGGA GCCTATCCTATCTTCTTTAAATGAGGCTGTTAAAGAATG
Mm_Sox9_pr1	AGCATTGGTGAGCTTTATCATCCTCTATGATTACTGACTGCGTCTATTTAGTGGAGC CGACCCTATCTTCTTTGGGAATAAACAGATAACATAG
Mm_Sox9_pr2	AGGTCTCAATGTTGGAGATGTCCTCTATGATTACTGACTGCGTCTATTTAGTGGAG CCGACCCTATCTTCTTTGTCAAACTCATTGACGTCGA
Mm_Sox9_pr3	GCATCTGAAACCTCTCATTTGTCCTCTATGATTACTGACTGCGTCTATTTAGTGGAG CCGACCCTATCTTCTTTGACTCAGTGCTCCTCACT
Mm_Gm14014_pr1	TCATTACTTGCTACTGTTGGGATTACTGACTGCGTCTATTTACTGTGATAGAATCCT TCTTGAGTTTATATGTCAATATGCACTCAAGGTCAC
Mm_Gm14014_pr2	CTCATGATTCCAGGGAGGATTACTGACTGCGTCTATTTACTGTGATAGAATCCTTCT TGAGTTTATATGTTCCACATCTCTTTATAACTATTTC
Mm_Gm14014_pr3	ATTTCATTTACTCACTGACGTTAGGATTACTGACTGCGTCTATTTACTGTGATAGAA TCCTTCTTGAGTTTATATGTCCAGAGTAGTACGGATCAAT
SCRINSHOT detection oligos	
Mm_Acta2_pr1	AGTGGACCUTCCTCTGTUGAAG [FITC]
Mm_Acta2_pr2	TCCTTCCUGATGTCAATAUCACACT [FITC]
Mm_Acta2_pr3	TAAACCTCUGTAGTUCCCATTCUGATAG [FITC]
Mm_Col1a2_pr1	TATUGAGAGATUTCAGAGTTGCAUCAAC [Cy5]
Mm_Col1a2_pr2	TATCUGAAATACTACUAATTAGCACCTTTGUG [Cy5]
Mm_Col1a2_pr3	GGCTGTTAAAGAAUGAATGCTGAATCUA [Cy5]
Mm_Sox9_pr1	CAGAUAACAUAGAGCATTGGUGAGC [Cy5]
Mm_Sox9_pr2	TGACGUCGAAGGUCTCAAUGT [Cy5]
Mm_Sox9_pr3	GCTCCUCACUGCATCUGAAAC [Cy5]
Mm_Gm14014_pr1	CTGTGAUAGAATCCTUCTTGAGTUTATATGT [FITC]

Supplementary Table S2

Length	1044
Identity	408/1044 (39,1%)
Similarity	408/1044 (39,1%)
Gaps	419/1044 (40,1%)
Score	1605,5

```
Wt1_76127          1 CCGAGGAGGGGCGG-CCCGCGTGGCTCAGGT-----------GAGCCCCC     38
                        |.||..|||.| .||||..||.|||||.           |||.|.||
Gm_14014           1 ---AAGAAAGGCAGTACCGCAAGGTTCAGGAAAAGAGGTCAGGAGACTCC     47

Wt1_76127         39 TGAGGGCA--GGGCAGGGCTGGG----CTGCACGGCCAC--------AGA     74
                     | .|..||  ...|||..|..||    |..||.|.|.|.        .||
Gm_14014          48 T-TGTACATCAATCAGCTCCTGGAAAACAACATGTCAATGAGTTAAGTGA     96

Wt1_76127         75 CCGCGGCCCAACCTC----GGC-------ACCCCGGAAGGCGGCCTG---    110
                     .|.||...||..|.|    ||.       .|||..|||.....||||
Gm_14014          97 ACACGAATCAGGCACATATGGATTGAATTTCCCTTGAAATACTCCTGGGG    146

Wt1_76127        111 ------------AGGGTGGTAGGGCGG-----------------------    125
                                 .||||||.|.||.||
Gm_14014         147 TTCAAGTTTTAAGGGGTGGAATGGAGGAGGGGTGAAGAATCTTGGGAAAA    196

Wt1_76127        126 -----TGCCCCGG-------------------------------AGGAAG    139
                          .|||.|.|                               ||.|||
Gm_14014         197 TCTGCAGCCACAGAATGTCTGAAAATACTTCTAAACTGTCGACCAGCAAG    246

Wt1_76127        140 GCTGCAGAGGTTCCAGATGGACTCCCGTGCAGTGCTGG---CAGTTG---    183
                     ||||||.|   |||.|.||..||  .|..||.||||||   |||.||
Gm_14014         247 GCTGCAAA---TCCTGCTGACCT--GGATCAATGCTGGGTCCAGCTGACC    291

Wt1_76127        184 ---GCGTGCTGTAGCGGCGGCGA----TGTCCC----GT-----------    211
                        ||.|.||.|||..|.|.|.|    ||.|.|    ||
Gm_14014         292 TTTGCTTTCTTTAGATGAGACCAGATCTGACACTGGAGTGACAGCCAAGG    341

Wt1_76127        212 --TTCCCGCCACTTGA-CCGATC-ACCTTCTCT--CAC-----------A    244
                       |||||.|| ||||| |.|..| .|.|||..|  |||           |
Gm_14014         342 ACTTCCCACC-CTTGATCTGCACTTCATTCCATGGCACTGAGGATAGAAA    390

Wt1_76127        245 GATCTGGGCT------TTCCAGAGGAGCTTCTTGG--GGAAAGTGG----    282
                     |.|||  |||      |.||...|.||||.|..||  ||.|..|||
Gm_14014         391 GGTCT--GCTAGAAAGTGCCTTTGAAGCTCCAGGGAAGGGAGCTGGGATT    438

Wt1_76127        283 ---TTCTG-----AAG--TTCTGT-AAAAACTTTGAAG----CCTGTTT-    316
                        |||.|     |||  ..|||| |.||.|.||.|||    ||.|.|.
Gm_14014         439 TATTTCAGTCAGAAAGCAAACTGTGAGAAGCATTCAAGGTGACCAGATAT    488

Wt1_76127        317 -----CTACTAGCACTGAAAACTG-AAG----TTCATCTGTGAAACAGAA    356
                          |.|.||||. ||....|.| |||    ||||||. |....||...
Gm_14014         489 GATGGCCAGTAGCT-TGTGTTCAGCAAGTCCATTCATCC-TTCTCCAACG    536

Wt1_76127        357 CCTT--------------TGCAGAGAAGGAACTACAGCAGTTTTGGAGTA    392
                     ||||              ||..||.|..|...||.|| ||.|.|||||.|
Gm_14014         537 CCTTCCTCCCTGGAATCATGAGGAAATAGTTATAAAG-AGATGTGGAGAA    585

Wt1_76127        393 CAGGAGACCACCTTC--ATCTTTGTAGCATGCTCA------GCTCTGGCT    434
                     .||     ||.||..  .||||.|.|.|||.||||      .|.|...||
Gm_14014         586 AAG-----CAACTGAGTTTCTTGGAACCATCCTCAAACAACACACATCCT    630

Wt1_76127        435 CG----CAGATCTTTGCA----TTCTGCGCTACTCAGAACAGCATAAACG    476
                     .|    |.|..|..||||    |.||.|.||.||....||. |||||| |
Gm_14014         631 TGTGTTCGGCACAGTGCAAATGTCCTCCACTCCTATAGACC-CATAAA-G    678

Wt1_76127        477 CTTTCAGTC--TTCCCAAGGC---TCTG----------------------    499
                     ..|..||.|  |.|.||||||   ||||
Gm_14014         679 TATGAAGGCAATGCGCAAGGCAGATCTGCAGTAGGATGGAAGAGTATGTG    728
```

```
Wt1_76127            500 -------------------AAATTATTCAGTCC-ATAGAGACTTATCAC-    528
                                            |..||.|||.|||| |||.|||  |.|||.
Gm_14014             729 TGAAAGAAAAGAAACAGTCATGTTTTTCTGTCCAATATAGA--TTTCATG    776

Wt1_76127            529 ----ATTGCCAAGCT--CATATAAT------------CTCC------TTA    554
                             .|.||....||  |||.|.||            ||.|      ||.
Gm_14014             777 ATTGTTAGCACTACTTACATCTGATTTGAGGTTGACACTGCAAGAGTTTT    826

Wt1_76127            555 CCGTGG-------------TCAGTTTC----ACACAAGT----AACATGA    583
                         ..||||             ||||||.|    |||||..|    |||.||.
Gm_14014             827 GAGTGGAGGATAAGTTACTTCAGTTACATATACACAGATTCTAAACTTGG    876

Wt1_76127            584 T---GACTTATCCTTATGC--------TGTCCTGGCCAATAT--------    614
                         |   .||..|.||||.|.|        ..||.|||..|||.|
Gm_14014             877 TTAATACCAAGCCTTTTACCTAGAAAGCATCATGGGTAATTTAAGATGAG    926

Wt1_76127            615 TAAAGT---ATTCTTTCCAAAT--AAAAC------AGCTTCTAATT----    649
                         .||.||   .|||.|..|.|||  .||||      ||...|.|..|
Gm_14014             927 AAAGGTGCATTTCCTCTCTAATGGGAAACCTGGTGAGAAACGAGATGTAT    976

Wt1_76127            650 --------------------------------------------   649

Gm_14014             977 TTCATTTGTTGTTGTTTTGTGCAGCATTTTACAGTCGAGCCAAC  1020
```

Uniprot ID	Gene	Short name	Subcellular location	Gene ontology
Q9CSS6	1810009A15Rik	LBH domain-containing 1	Nucleus	-
Q9D809	2200002D01Rik	2200002D01Rik	Nucleus	-
P68134	Acta1	Actin Alpha 1	Cytosol, cytoskeleton	Cell motility
P62737	Acta2	Actin Alpha 2	Cytosol, cytoskeleton	Cell motility
Q8BFZ3	Actbl2	Actin Beta Like 2	Nucleus, cytoskeleton, extracellular	Cell motility
P68033	Actc1	Actin Alpha Cardiac Muscle 1	Cytosol, cytoskeleton	Cell motility
P63268	Actg2	Actin Gamma 2	Cytosol, cytoskeleton	Cell motility
Q7TPR4	Actn1	Actinin Alpha 1	Cytosol, cytoskeleton	Actin cytoskeleton organization
Q9JI91	Actn2	Actinin Alpha 2	Cytosol, cytoskeleton	Actin cytoskeleton organization
O88990	Actn3	Actinin Alpha 3	Cytosol, cytoskeleton, extracellular, plasma membrane	Actin cytoskeleton organization
P57780	Actn4	Actinin Alpha 4	Cytosol, nucleus, cytoskeleton	Actin cytoskeleton organization
P55264	Adk	Adenosine Kinase	Cytosol, nucleus, plasma membrane	Phosphorylation
P45376	Akr1b1	Aldo-Keto Reductase Family 1 Member B	Cytosol, nucleus	Enzymatic activity, NADPH-dependent reduction
P05064	Aldoa	Aldolase, Fructose-Bisphosphate A	Cytosol	Glycolysis and gluconeogenesis, scaffolding protein
A6ZI47	Aldoart2	Aldolase, Fructose-Bisphosphate A	Cytosol	Glycolysis and gluconeogenesis, scaffolding protein
Q9WV07	Aloxe3	Arachidonate Lipoxygenase 3	Cytosol	Catalitic activity
P10107	Anxa1	Annexin A1	Cytosol, endosome, nucleus, extracellular, plasma membrane	Actin cytoskeleton organization, innate immune response
O35639	Anxa3	Annexin A3	Extracellular, plasma membrane	Inhibitor of phospholipase A2, anti-coagulant properties
P48036	Anxa5	Annexin A5	Cytosol, extracellular	Anticoagulant protein
P84078	Arf1	ADP Ribosylation Factor 1	Golgi apparatus	Protein trafficking, microbial infection, actin filament organization
Q8BSL7	Arf2	ADP Ribosylation Factor 2	-	Intracellular protein transport
P61205	Arf3	ADP Ribosylation Factor 3	Golgi apparatus, cytosol, extracellular, plasma membrane	Protein trafficking, GTP-binding protein

Uniprot ID	Gene	Short name	Subcellular location	Gene ontology
P61750	Arf4	ADP Ribosylation Factor 4	Cytosol, plasma membrane	Activation of phospholipase D activity, protein trafficking, GTP-binding protein
P84084	Arf5	ADP Ribosylation Factor 5	Cytosol, extracellular, plasma membrane	Protein trafficking, GTP-binding protein
Q99PT1	Arhgdia	Rho GDP Dissociation Inhibitor Alpha	Cytosol, cytoskeleton	Rho proteins homeostasis, cell motility regulation.
Q8K0N8	Bms1	BMS1 Ribosome Biogenesis Factor	Nucleus	Molecular switch during maturation of the 40S ribosomal subunit
Q3UGC3	Cald1	Caldesmon 1	Cytosol, cytoskeleton, plasma membrane	Actin- and myosin-binding, regulation of actomyosin interactions
P14211	Calr	Calreticulin	Cytosol, endoplasmic reticulum, nucleus, extracelular	Calcium-binding chaperone, oocyte maturation, maternal gene expression regulation
P35564	Canx	Calnexin	Endoplasmic reticulum, extracelular	Calcium-binding protein, thymocyte maturation, endocytosis at the synapse
P47753	Capza1	Capping Actin Protein Of Muscle Z-Line Subunit Alpha 1	Cytosol, cytoskeleton	F-actin-capping proteins, epithelial cell junctions
P47754	Capza2	Capping Actin Protein Of Muscle Z-Line Subunit Alpha 2	Cytosol	F-actin-capping proteins
O89094	Casp14	Caspase 14	Cytosol	Non-apoptotic caspase, epidermal differentiation
P11983	Cct2	Chaperonin Containing TCP1 Subunit 2	Cytosol, cytoskeleton	Chaperonin-containing T-complex (TRiC)
P80316	Cct5	Chaperonin Containing TCP1 Subunit 5	Cytosol, cytoskeleton	Chaperonin-containing T-complex (TRiC)
P27790	Cenpb	Centromere Protein B	Nucleus	Interacts with centromeric heterochromatin
Q62036	Cep131	Centrosomal Protein 131	Cytoskeleton	Component of centriolar satellites, regulate cilia/flagellum formation
P18760	Cfl1	Cofilin 1	Cytosol, nucleus	Binds to F-actin, regulation of actin dynamics
P45591	Cfl2	Cofilin 2	Cytosol, nucleus, cytoskeleton, extracellular	Actin polymerization and depolymerization, muscle maintenance
Q921W0	Chmp1a	Charged Multivesicular Body Protein 1A	Cytosol, endosome, nucleus, extracellular, cytoskeleton	Transport complex III (ESCRT-III), chromosome condensation, autophagosome maduration
Q9JI95	Cpn10-rs1	CPN10-like protein	Mitochondrion	Chaperone cofactor-dependent protein refolding
Q8R555	Crtac1	Cartilage Acidic Protein 1	Extracellular	Axonal fasciculation, chondrogenic differentiation and by BMP4
P16381	D1Pas1	DEAD-Box Helicase 3 X-Linked	Cytosol, nucleus, cytoskeleton, plasma membrane	Multifunctional ATP-dependent RNA helicase, transcription regulation, regulation of translation initiation, innate immunity

Uniprot ID	Gene	Short name	Subcellular location	Gene ontology
Q501J6	Ddx17	DEAD-Box Helicase 17	Nucleus	Pre-mRNA splicing, alternative splicing, ribosomal RNA processing and miRNA processing, transcription regulation
Q9JIK5	Ddx21	DExD-Box Helicase 21	Cytosol, nucleus	Sensor of the transcriptional status of both RNA polymerase (Pol) I and II, chromatin remodeling, innate immune response
Q8VDW0	Ddx39a	DExD-Box Helicase 39A	Nucleus	Pre-mRNA splicing
Q9Z1N5	Ddx39b	DExD-Box Helicase 39B	Nucleus	Nuclear export of spliced and unspliced mRNA, transcription elongation and genome stability
Q62167	Ddx3x	DEAD-Box Helicase 3 X-Linked	Cytosol, nucleus, cytoskeleton, plasma membrane	Multifunctional ATP-dependent RNA helicase
Q62095	Ddx3y	DEAD-Box Helicase 3 Y-Linked	Cytosol, nucleus	ATP-dependent RNA helicase, immune response, cell differentiation
Q61656	Ddx5	DEAD-Box Helicase 5	Nucleus	Alternative regulation of pre-mRNA splicing
Q99MJ9	Ddx50	DExD-Box Helicase 50	Nucleus	Catalitic activity
Q8K4L0	Ddx54	DEAD-Box Helicase 54	Nucleus	RNA-dependent ATPase activity, represses the transcriptional activity of nuclear receptors
O35286	Dhx15	DEAH-Box Helicase 15	Nucleus	mRNA processing, antiviral innate immunity
Q8VHK9	Dhx36	DEAH-Box Helicase 36	Cytosol, nucleus	Genomic integrity, gene expression regulations, sensor to initiate antiviral responses
Q6P5D3	Dhx57	DExH-Box Helicase 57	Nucleus	ATP-binding RNA helicase
B1AZP2	Dlgap4	DLG Associated Protein 4	Nucleus, plasma membrane	Molecular organization of synapses and neuronal cell signaling, adapter protein linking ion channel to the subsynaptic cytoskeleton
Q9QYJ0	Dnaja2	DnaJ homolog subfamily A member 2	Cytosol	Co-chaperone of Hsc70
Q61508	Ecm1	Extracellular Matrix Protein 1	Extracellular	Negative regulator of bone mineralization, endothelial proliferation, angiogenesis, regulate MMP9 proteolytic activity
P57776	Eef1d	Eukaryotic Translation Elongation Factor 1 Delta	Cytosol, nucleus	Induction of heat-shock-responsive genes, ribosomal regulation
Q9D8N0	Eef1g	Eukaryotic Translation Elongation Factor 1 Gamma	Cytosol, nucleus, extracellular	Anchoring the complex to other cellular components
A0JP43	Efcab5	EF-Hand Calcium Binding Domain 5	Cytosol, nucleus	Enables calcium ion binding, metal ion binding
Q6ZWX6	Eif2s1	Eukaryotic Translation Initiation Factor 2 Subunit Alpha	Cytosol	Nucleic acid binding, RNA binding, trasnlation iniciation factor activity, protein binding, ribosome binding
Q9Z0N1	Eif2s3x	Eukaryotic Translation Initiation Factor 2 Subunit Gamma	Cytosol	tRNA binding, nucleotide binding, translation iniciation factor activity, GTPase activity, protein binding

Uniprot ID	Gene	Short name	Subcellular location	Gene ontology
Q9Z0N2	Eif2s3y	Eukaryotic Translation Initiation Factor 2 Subunit Gamma	Cytosol	tRNA binding, nucleotide binding, translation iniciation factor activity, GTPase activity, protein binding
Q8R1B4	Eif3c	Eukaryotic Translation Initiation Factor 3 Subunit C	Cytosol	RNA binding, translation iniciation factor activity, protein binding, ribosome binding
P70372	Elavl1	ELAV Like RNA Binding Protein 1	Cytosol, nucleus	Nucleic acid binding, RNA, RNAm, protein binding, 3'-UTR-mediated mRNA stabilization
P17182	Eno1	Enolase 1	Cytosol, nucleus, extracellular, plasma membrane	Magnesium ion binding, RNA pol II transcription regulatory, DNA binding, canonical glucolysis
Q9D952	Evpl	Envoplakin	Cytosol, cytoskeleton, plasma membrane	Structural molecule activity, protein binding, intermediate filament binding, cadherin binding
P26040	Ezr	Ezrin	Cytosol, cytoskeleton, endosome, plasma membrane, nucleus	RNA binding, actin binding, protein binding
Q8BUM6	Fam163b	Family With Sequence Similarity 163 Member B	Endoplasmic reticulum, plasma membrane	Protein binding
Q8BP22	Fam92a	CBY1 Interacting BAR Domain Containing 1	Mitochondrion, nucleus, cytoskeleton	Protein binding, phospholipid binding, cilium assembly, inner mitochondrial membrane organization
Q99M01	Fars2	Phenylalanyl-TRNA Synthetase 2, Mitochondrial	Mitochondrion	tRNA binding, nucleotide binding, aminoacyl-tRNA ligase activity, phenylalanine-tRNA ligase activity
Q8BTM8	Flna	Filamin A	Cytosol, nucleus, cytoskeleton, plasma membrane	G protein-coupled receptor binding, RNA binding, actin binding, protein kinase C binding
Q8VHX6	Flnc	Filamin C	Cytosol, plasma membrane	Cytoskeletal protein binding
A2APV2	Fmnl2	Formin Like 2	Cytosol	Actin binding, small GTPase binding
P35922	Fmr1	Fragile X Messenger Ribonucleoprotein 1	Cytosol, nucleus	G-quadruplex RNA binding, DNA binding, RNA binding
Q61553	Fscn1	Fascin Actin-Bundling Protein 1	Cytosol, cytoskeleton, plasma membrane	RNA binding, actin binding, protein binding, protein-macromolecule adaptor activity
Q61584	Fxr1	FMR1 Autosomal Homolog 1	Cytosol, nucleus	Nucleic acid binding, mRNA destabilization
Q9WVR4	Fxr2	FMR1 Autosomal Homolog 2	Cytosol	Nucleic acid binding, mRNA destabilization
P16858	Gapdh	Glyceraldehyde-3-Phosphate Dehydrogenase	Cytosol, nucleus, cytoskeleton, extracellular, plasma membrane	Nucleotide binding, phosphorylating activity, microtubule binding, oxidoreductase activity
Q64467	Gapdhs	Glyceraldehyde-3-Phosphate Dehydrogenase, Spermatogenic	Cytosol, nucleus	Phosphorylating activity, protein binding, oxidoreductase activity, peptidyl-cysteine S-nitrosylase activity
Q9CPV4	Glod4	Glyoxalase Domain Containing 4	Mitochondrion, extracellular	Cadherin binding

Uniprot ID	Gene	Short name	Subcellular location	Gene ontology
Q9QUH0	Glrx	Glutaredoxin	Cytosol	Protein binding, phospholipid binding, glutathione disulfide oxidoreductase activity, transferase activity
F6YVP7	Gm10260	40S ribosomal protein S18	Cytosol	Translation
A2AMD0	Gm12666	Predicted gene 12666	Nucleus	-
E9PZF0	Gm20390	Nucleoside diphosphate kinase	Desmosome	CTP, GTP and UTP biosynthetic process, phosphorylation
W4VSN7	Gm3550	60S ribosomal protein L29	Nucleus	Cytoplasmic translation
S4R1W1	Gm3839	Glyceraldehyde-3-phosphate dehydrogenase	Nucleus, cytoskeleton	Glucose metabolic process
A0A0A6YW67	Gm8797	Predicted pseudogene 8797	-	-
P68040	Gnb2l1	Receptor For Activated C Kinase 1	Cytosol, nucleus, plasma membrane	Protein binding, RNA binding, ion channel inhibitor activity, translation regulator activity
Q99LH1	Gnl2	G Protein Nucleolar 2	Nucleus	RNA binding, GTPase activity, ribosome biogenesis
Q8CI11	Gnl3	Guanine nucleotide-binding protein-like 3	Nucleus	Protein binding, RNA binding, GTP binding
P05201	Got1	Aspartate aminotransferase	Cytosol	Catalytic activity
P13020	Gsn	Gelsolin	Cytosol, cytoskeleton, extracellular, plasma membrane	Actin and myosin binding, calcium ion binding, catalytic binding
P13745	Gsta1	Glutathione S-Transferase Alpha 1	Cytosol	Catalytic activity
P30115	Gsta3	Glutathione S-Transferase Alpha 3	Cytosol	Glutathione transferase activity, transferase activity
P24472	Gsta4	Glutathione S-Transferase Alpha 4	Cytosol	Catalytic activity, protein binding
Q99ME9	Gtpbp4	GTP Binding Protein 4	Cytosol, nucleus	Maduration of LSU-rRNA from tricistronic rRNA transcript, cell migration, collagen binding
P10922	H1f0	H1.0 Linker Histone	Nucleus, cytoskeleton	DNA, RNA and protein binding, structural constituent of chromatin
Q80ZM5	H1fx	H1.10 Linker Histone	Nucleus	DNA, RNA and protein binding, structural constituent of chromatin
Q8R1M2	H2afj	H2A.J Histone	Extracellular	DNA binding, structural constituent of chromatin, protein heterodimerization activity
Q3THW5	H2afv	H2A.Z Variant Histone 2	Nucleus	DNA binding, structural constituent of chromatin, protein heterodimerization activity
P27661	H2afx	H2A.X Variant Histone	Nucleus, cytoskeleton	DNA, protein and histone binding, enzyme binding, structural constituent of chromatin, protein heterodimerization activity

Uniprot ID	Gene	Short name	Subcellular location	Gene ontology
P0C0S6	H2afz	H2A.Z Variant Histone 1	Nucleus	RNA polymerase II cis-regulatory region, DNA, RNA, protein and chromatin binding, cellular response to estradiol and insulin stimulus
P01942	Hba2	Hemoglobin Subunit Alpha 2	Cytosol, extracellular	Peroxidase activity, oxygen carrier activity, metal ion binding
P01942	Hba	Hemoglobin Subunit Alpha 1	Cytosol, extracellular	Peroxidase activity, oxygen carrier activity, metal ion binding
P43275	Hist1h1a	H1.1 Linker Histone, Cluster Member	Nucleus	DNA, RNA and protein binding, structural constituent of chromatin
P43276	Hist1h1b	H1.5 Linker Histone, Cluster Member	Nucleus	DNA, RNA and protein binding, structural constituent of chromatin
P15864	Hist1h1c	H1.2 Linker Histone, Cluster Member	Nucleus	DNA, RNA and protein binding, structural constituent of chromatin
P43277	Hist1h1d	H1.3 Linker Histone, Cluster Member	Nucleus	DNA, RNA and protein binding, structural constituent of chromatin
P43274	Hist1h1e	H1.4 Linker Histone, Cluster Member	Nucleus	DNA, RNA and protein binding, structural constituent of chromatin
Q8CGP4	Hist1h2aa	H2A Clustered Histone 1	Nucleus	DNA binding, structural constituent of chromatin
Q8CGP5	Hist1h2af	H2A Clustered Histone 11	Nucleus	DNA and protein binding, structural constituent of chromatin
Q8CGP6	Hist1h2ah	H2A Clustered Histone 12	Nucleus	DNA binding, structural constituent of chromatin
Q8CGP7	Hist1h2ak	H2A Clustered Histone 15	Nucleus	DNA and protein binding, structural constituent of chromatin
P70696	Hist1h2ba	H2B Clustered Histone 1	Nucleus	DNA binding, structural constituent of chromatin
Q64475	Hist1h2bb	H2B Clustered Histone 3	Nucleus	DNA and protein binding, structural constituent of chromatin
Q6ZWY9	Hist1h2bc	H2B Clustered Histone 4	Nucleus, extracellular	DNA and protein binding, structural constituent of chromatin
P10853	Hist1h2bf	H2B Clustered Histone 7	Nucleus, extracellular	DNA and protein binding, structural constituent of chromatin
Q64478	Hist1h2bh	H2B Clustered Histone 9	Nucleus	DNA and protein binding, structural constituent of chromatin, ubiquitin-like protein ligase binding
Q8CGP1	Hist1h2bk	H2B Clustered Histone 12	Nucleus, extracellular	DNA and protein binding, structural constituent of chromatin, antibacterial response
P10854	Hist1h2bm	H2B Clustered Histone 14	Nucleus	DNA binding, structural constituent of chromatin,
Q8CGP2	Hist1h2bp	H2B Clustered Histone 17	Nucleus	DNA and protein binding, structural constituent of chromatin
Q6GSS7	Hist2h2aa1	H2A Clustered Histone 18	Nucleus	DNA and protein binding, structural constituent of chromatin
Q64522	Hist2h2ab	H2A Clustered Histone 21	Nucleus	DNA and protein binding, structural constituent of chromatin

Uniprot ID	Gene	Short name	Subcellular location	Gene ontology
Q64523	Hist2h2ac	H2A Clustered Histone 20	Nucleus	DNA and protein binding, structural constituent of chromatin
Q64525	Hist2h2bb	H2B Histone Pseudogene 2	-	-
Q64524	Hist2h2be	H2B Clustered Histone 21	Nucleus, extracellular	DNA and protein binding, structural constituent of chromatin, antibacterial response
Q8BFU2	Hist3h2a	H2A Clustered Histone 25	Nucleus	DNA and protein binding, structural constituent of chromatin
Q9D2U9	Hist3h2ba	H2B Clustered Histone 27, Pseudogene	-	-
Q8CGP0	Hist3h2bb	H2B Clustered Histone 26	Nucleus	DNA binding, structural constituent of chromatin
P30681	Hmgb2	High Mobility Group Box 2	Nucleus, extracellular	Four-way junction DNA binding, transcription cis-regulatory region binding, cell chemotaxis
O88569	Hnrnpa2b1	Heterogeneous Nuclear Ribonucleoprotein A2/B1	Nucleus, extracellular	Nucleic acid binding, G-quadruplez DNA unwinding, miRNA transport, mRNA export from nucleus, Mrna splicing
Q9Z204	Hnrnpc	Heterogeneous Nuclear Ribonucleoprotein C	Cytosol, nucleus, cytoskeleton	Nucleic acid binding, 3'-UTR mediated mRNA stabilization, mRNA splicing
Q60668	Hnrnpd	Heterogeneous Nuclear Ribonucleoprotein D	Nucleus	Nucleuc acid binding, protein binding, minor groove of adenine-thymine-rich DNA binding, chromatin binding, 3'-UTR mediated mRNA destabilization
Q9Z2X1	Hnrnpf	Heterogeneous Nuclear Ribonucleoprotein F	Nucleus	Nucleic acid binding, mRNA processing, RNA splicing
O35737	Hnrnph1	Heterogeneous Nuclear Ribonucleoprotein H1	Cytosol, nucleus	Nucleic acid binding, cellular response to IL-7, RNA splicing
P70333	Hnrnph2	Heterogeneous Nuclear Ribonucleoprotein H2	Nucleus	Nucleic acid binding, protein binding, regulation of RNA splicing
Q9D0E1	Hnrnpm	Heterogeneous Nuclear Ribonucleoprotein M	Nucleus	Nucleic acid binding, protein binding, induction of cytokines, alternative mRNA, protein localization to nucleus
Q8VEK3	Hnrnpu	Heterogeneous Nuclear Ribonucleoprotein U	Cytosol, nucleus, cytoskeleton	Nucleic acid binding, RNA pol II binding, adaptative thermogenesis, cardiac muscle cell development, cell cycle
Q8VDM6	Hnrnpul1	Heterogeneous Nuclear Ribonucleoprotein U Like 1	Nucleus	RNA, protein and enzyme binding
Q3TEA8	Hp1bp3	Heterochromatin Protein 1 Binding Protein 3	Nucleus	DNA, protein and chromatin binding, cellular response to hipoxia, regulation of cell proliferation, regulation of nucleus size
Q0VGQ1	Hsd17b12	Hydroxysteroid 17-Beta Dehydrogenase 12	Endoplasmic reticulum	Fibronectin binding, estradiol 17-beta-dehydrogenase [NAD(P)] activity, protein binding, collagen binding, heparin binding
P48722	Hspa4	Heat Shock Protein Family A (Hsp70) Member 4	Cytosol, nucleus, extracellular	Nucleotide binding, protein binding, ATP binding, ATP-dependent protein folding chaperone

Uniprot ID	Gene	Short name	Subcellular location	Gene ontology
P63038	Hspe1	Heat Shock Protein Family E (Hsp10) Member 1	Mitochondrion	RNA and protein binding, ATP binding, metal ion binding, protein folding chaperone, apoptotic mitochondrial changes, B cell proliferation
Q61699	Hsph1	Heat Shock Protein Family H (Hsp110) Member 1	Cytosol, nucleus	Nucleotide binding, protein binding, ATP binding, alpha-tubulin binding, adenyl-nucleotide exchange factor activity, NK T cell activation, neuron apoptotic process
O88844	Idh1	Isocitrate Dehydrogenase (NADP(+)) 1	Cytosol, peroxisome	Magnesium ion binding, protein binding, isocitrate dehydrogenase (NADP+) activity, oxidoreductase activity
Q5SF07	Igf2bp2	Insulin Like Growth Factor 2 MRNA Binding Protein 2	Cytosol, nucleus	Nucleic acid binding, RNA binding, RNAm binding, thermogenesis, regulation of translation
Q9CXY6	Ilf2	Interleukin Enhancer Binding Factor 2	Nucleus	DNA, RNA and protein binding
Q9Z1X4	Ilf3	Interleukin Enhancer Binding Factor 3	Nucleus, mitochondrion	DNA and RNA binding, regulation of transcription
Q8VHZ7	Imp4	IMP U3 Small Nucleolar Ribonucleoprotein 4	Nucleus	Protein binding, RNAr binding, snoRNA binding
P24547	Impdh2	Inosine Monophosphate Dehydrogenase 2	Cytosol, nucleus	DNA and RNA binding, catalytic activity, IMP dehydrogensase activity, cellular response to IL-4, circadian rhythm
Q8CHT3	Ints5	Integrator Complex Subunit 5	Nucleus	Protein binding
Q62470	Itga3	Integrin Subunit Alpha 3	Extracellular, plasma membrane	Fibronectin binding, protease binding, integrin binding, collagen binding, cell adhesion
Q80WE4	Kif20b	Kinesin Family Member 20B	Cytosol, nucleus, cytoskeleton	Nucleotide binding, cytoskeletal motor activity, cell cycle
Q61771	Kif3b	Kinesin Family Member 20B	Cytosol, nucleus, cytoskeleton	Nucleotide binding, cytoskeletal motor activity, anterograde dendritic transport of neurotransmitter receptor complex, cilium assembly
P02535	Krt10	Keratin 10	Cytosol, nucleus, cytoskeleton	Structural molecule activity, protein binding, structural constituent of skin epidermis, intermediate filament organization
Q8VCW2	Krt25	Keratin 25	Cytosol, nucleus, extracellular	Structural molecule activity, protein binding, citoskeleton organization, ephitelial cell differentiation
Q9Z320	Krt27	Keratin 27	Cytosol, extracellular	Structural molecule activity, protein binding, epithelial cell differentiation, intermediate filament organization
A6BLY7	Krt28	Keratin 28	Cytosol, extracellular	Structural molecule activity, protein binding, epithelial cell differentiation, intermediate filament organization
P50446	Krt6a	Keratin 6A	Cytosol, nucleus	Structural constituent of cytoskeleton, protein binding, structural constituent of skin epidermis, antimicrobial humoral response
Q9Z331	Krt6b	Keratin 6B	Cytosol	Structural constituent of cytoskeleton, protein binding, structural constituent of skin epidermis

Uniprot ID	Gene	Short name	Subcellular location	Gene ontology
Q8BGZ7	Krt75	Keratin 75	Cytosol	Structural constituent of cytoskeleton, protein binding, structural constituent of skin epidermis hematopoietic progenitor cell differentiation
Q8VED5	Krt79	Keratin 79	Cytosol	Enzyme binding, protein binding, structural constituent of skin epidermis
Q9Z2T6	Krt85	Keratin 85	Cytosol, extracellular, cytoskeleton	Structural molecule activity, protein binding, structural constituent of skin epidermis
Q80XH1	Kxd1	KxDL Motif Containing 1	Cytosol	Lysosome localization, vesicle-mediated transport, organelle transport along microtubule
Q9CPY7	Lap3	Leucine Aminopeptidase 3	Cytosol, nucleus	Aminopeptidase activity, carboxypeptidase activity, peptidase activity, metalloexopeptidase activity
Q05CL8	Larp7	La Ribonucleoprotein 7, Transcriptional Regulator	Nucleus	Nucleic acid binding, protein binding, U6 snRNA binding, cell differentiation, mRNA processing
P06151	Ldha	Lactate Dehydrogenase A	Cytosol, nucleus, extracellular	Catalytic activity, protein binding, cellular response to extracellular stimulus
P00342	Ldhc	Lactate Dehydrogenase C	Cytosol	Catalytic activity
Q9CYI4	Luc7l	LUC7 Like	Nucleus	RNAm and protein binding, negative regulation of striated muscle tissue development
Q7TNC4	Luc7l2	LUC7 Like 2, Pre-MRNA Splicing Factor	Nucleus	RNA, protein and enzyme binding
Q5SUF2	Luc7l3	LUC7 Like 3 Pre-MRNA Splicing Factor	Nucleus	DNA, RNA and protein binding
P17897	Lyz1	Lysozyme	Extracellular	Catalytic activity, defense response to bacterium
Q66L42	Map3k10	Mitogen-Activated Protein Kinase Kinase Kinase 10	Cytosol	Nucleotide binding, transcription corepressor activity, protein serine/threonine kinase activity, JUN kinase kinase kinase activity
P26645	Marcks	Myristoylated Alanine Rich Protein Kinase C Substrate	Cytoskeleton	Actin binding, protein kinase C binding, calmodulin binding, apoptotic process
Q811L6	Mast4	Microtubule Associated Serine/Threonine Kinase Family Member 4	Cytosol	Nucleotide binding, magnesium binding, metalloendopeptidase activity, protein kinase activity, cytoskeleton organization
G3UVW1	Mpv17	Mitochondrial Inner Membrane Protein MPV17	Peroxisome, mitochondrion	Protein binding, channel activity
P26041	Msn	Moesin	Cytosol, nucleus, cytoskeleton, plasma membrane	RNA and protein binding, signaling receptor binding, structural constituent of cytoskeleton, establishment of epithelial cell apical/basal polarity, immunological synapse formation
Q7TPV4	Mybbp1a	MYB Binding Protein 1a	Nucleus	Cellular response to glucose starvation, chromatin remodeling, circadian regulation of gene expression, apoptotic regulation by p53, DNA and RNA binding, transcription factor binding

Uniprot ID	Gene	Short name	Subcellular location	Gene ontology
P05977	Myl1	Myosin Light Chain 1	Cytosol, cytoskeleton	Calcium ion binding, structural constituent of muscle
D3Z249	Myl12a	Myosin Light Chain 12A	Cytosol	Calcium ion binding, protein binding, myosin heavy chain binding, metal ion binding, protein localization to plasma membrane
Q3THE2	Myl12b	Myosin Light Chain 12B	Cytosol	Calcium ion binding, protein binding, myosin heavy chain binding, metal ion binding, regulation of cell shape
Q9CQ19	Myl9	Myosin Light Chain 9	Cytosol, cytoskeleton	Calcium ion binding, protein binding, myosin heavy chain binding, metal ion binding, structural constituent of muscle
E9PV66	Myo18b	Myosin XVIIIB	Nucleus, cytoskeleton	Nucleotide binding, cytoskeletal motor activity, actin binding, ATP binding, cardiac muscle cell development, vasculogenesis
P09405	Ncl	Nucleolin	Nucleus	Nucleic acid binding, obsolete protein C-terminus binding, angiogenesis, cellular response to epidermal growth factor stimulus
P15532	Nme1	NME/NM23 Nucleoside Diphosphate Kinase 1	Cytosol, nucleus, plasma membrane	Cell proliferation, differentiation and development, signal transduction, G protein-coupled receptor endocytosis, gene expression
Q01768	Nme2	NME/NM23 Nucleoside Diphosphate Kinase 2	Cytosol, nucleus	DNA and protein binding, kinase activity, transferase activity
Q9CPT5	Nop16	NOP16 Nucleolar Protein	Nucleus	Ribosomal large subunit biogenesis
Q61937	Npm1	Nucleophosmin 1	Nucleus, cytoskeleton	Ribosome biogenesis, centrosome duplication, protein chaperoning, histone assembly, cell proliferation, regulation of tumor suppressors p53/TP53 and ARF
D3Z2Y0	Nrros	Negative Regulator Of Reactive Oxygen Species	Endoplasmic reticulum, plasma membrane	Protein binding, growth factor binding, transforming growth factor beta binding
P0C646	Olfr67	Olfactory Receptor Family 52 Subfamily Z Member 1 Pseudogene	Plasma membrane	G protein-coupled receptor activity, olfactory receptor activity
Q3B7Z2	Osbp	Oxysterol Binding Protein	Golgi apparatus, cytosol, endoplasmic reticulum, nucleus	Protein binding, lipid binding, sterol transporter activity
P09103	P4hb	Prolyl 4-Hydroxylase Subunit Beta	Cytosol, endoplasmic reticulum, extracellular, plasma membrane	RNA binding, protein disulfide isomerase activity, actin and integrin binding, procollagen-proline 4-dioxygenase activity
P29341	Pabpc1	Poly(A) Binding Protein Cytoplasmic 1	Cytosol, nucleus	RNA, mRNA and protein binding
P60335	Pcbp1	Poly (RC) Binding Protein 1	Cytosol, nucleus	DNA-binding transcription factor activity, RNA polymerase II-specific
Q14690	Pdcd11	Programmed Cell Death 11	Nucleus	RNA and protein binding, NF-KB binding
P27773	Pdia3	Protein Disulfide Isomerase Family A Member 3	Endoplasmic reticulum, extracellular	RNA binding, protein disulfide isomerase activity, cysteine-type endopeptidase activity, phospholipase C activity
P62962	Pfn1	Profilin 1	Cytosol, nucleus, cytoskeleton	Actin binding, adenyl-nucleotide exchange factor activity, phosphotyrosine residue binding, actin cytoskeleton organization

Uniprot ID	Gene	Short name	Subcellular location	Gene ontology
Q9DBJ1	Pgam1	Phosphoglycerate Mutase 1	Cytosol	Catalytic activity
P83870	Phf5a	PHD Finger Protein 5A	Nucleus	Transcriptional elongation by RNA pol II, regulation of development, maintenance of embryonic stem cell (ESC) pluripotency
Q61753	Phgdh	Phosphoglycerate Dehydrogenase	Cytosol	Phosphoglycerate dehydrogenase activity, electron transfer activity
Q8BWR2	Pithd1	PITH Domain Containing 1	Nucleus	Megakaryocyte differentiation
P52480	Pkm	Pyruvate Kinase M1/2	Cytosol, nucleus, mitochondrion, extracellular	Nucleotide binding, magnesium binding, transcription coactivator activity, RNA binding
Q9Z280	Pld1	Phospholipase D1	Golgi apparatus, endosome, plasma membrane, lysosome	Catalytic activity, phospholipase D activity, hydrolase activity, membrane trafficking, regulation of mitosis
Q8K4L4	Pof1b	POF1B Actin Binding Protein	Cytoskeleton	Organization of epithelial monolayers by regulating the actin cytoskeleton, ovary development
P17742	Ppia	Peptidylprolyl Isomerase A	Cytosol, nucleus, extracellular	RNA and protein binding, integrin binding, cyclosporin A binding, chemotactic effect on leukocytes, activates endothelial cells (ECs) in a pro-inflammatory manner, apoptosis in Ecs
O08807	Prdx4	Peroxiredoxin 4	Extracellular	Activation of NF-kappa-B in the cytosol, cell protection against oxidative stress, thioredoxin peroxidase activity
Q9R1P4	Psma1	Proteasome 20S Subunit Alpha 1	Nucleus, cytoskeleton	Immune system process, proteolytic degradation, ATP-dependent degradation of ubiquitinated proteins
O70435	Psma3	Proteasome 20S Subunit Alpha 3	Cytosol, nucleus	Protein binding, ubiquitin protein ligase binding
Q9R1P0	Psma4	Proteasome 20S Subunit Alpha 4	Cytosol, nucleus	Proteolytic degradation
Q9Z2U0	Psma7	Proteasome 20S Subunit Alpha 7	Cytosol, nucleus	Proteolytic degradation, transactivation function of HIF-1A
Q9CWH6	Psma8	Proteasome 20S Subunit Alpha 8	Cytosol	Spermatoproteasome, cell differentiation, meiotic cell cycle
P26350	Ptma	Prothymosin Alpha	Cytosol, nucleus	DNA, protein and histone binding, immune function
O54724	Ptrf	Caveolae Associated Protein 1	Nucleus, mitochondrion, plasma membrane	Caveolae formation and organization, RNA and protein binding
O35295	Pura	Purine Rich Element Binding Protein A	Nucleus	Initiation of DNA replication and in recombination
Q5SW87	Rab1	RAB1A, Member RAS Oncogene Family	Golgi apparatus, cytosol	GTPase activity, intracellular membrane trafficking, clel adhesion and cell migration, autophagosome assembly
P61027	Rab10	RAB10, Member RAS Oncogene Family	Golgi apparatus, cytosol, endosome, endoplasmic reticulum	GTPase activity, intracellular membrane trafficking, cell adhesion and cell migration, autophagosome assembly
Q9DD03	Rab13	RAB13, Member RAS Oncogene Family	Golgi apparatus, cytosol, endosome, plasma membrane	GTPase activity, intracellular membrane trafficking

Uniprot ID	Gene	Short name	Subcellular location	Gene ontology
Q91V41	Rab14	RAB14, Member RAS Oncogene Family	Cytosol, endosome, plasma membrane	Membrane trafficking, GTPase activity
Q8K386	Rab15	RAB15, Member RAS Oncogene Family	Endosome	Regulation of aspects of synaptic vesicle membrane flow within the nerve terminal
P62821	Rab1A	RAB1A, Member RAS Oncogene Family	Golgi apparatus, cytosol	GTPase activity, intracellular membrane trafficking
Q9D1G1	Rab1b	RAB1B, Member RAS Oncogene Family	Golgi apparatus, cytosol	GTPase activity, intracellular membrane trafficking
Q8BHD0	Rab39a	RAB39A, Member RAS Oncogene Family	Cytosol	GTPase activity, vesicular trafficking, maturation and acidification of phagosomes
P35279	Rab6a	RAB6A, Member RAS Oncogene Family	Golgi apparatus, cytosol	Regulator of COPI-independent retrograde transport from the Golgi apparatus towards the endoplasmic reticulum (ER)
P61294	Rab6b	RAB6B, Member RAS Oncogene Family	Golgi apparatus	GTPase activity, intracellular membrane trafficking, hydrolase activity
P55258	Rab8a	RAB8A, Member RAS Oncogene Family	Golgi apparatus, cytosol, endosome, cytoskeleton	GTPase activity, intracellular membrane trafficking, hydrolase activity
P61028	Rab8b	RAB8B, Member RAS Oncogene Family	Peroxisome	GTPase activity, intracellular membrane trafficking, signaling receptor binding
P63001	Rac1	Rac Family Small GTPase 1	Golgi apparatus, cytosol, endosome, nucleus, extracellular, plasma membrane	GTPase activity, regulate cellular responses such as secretory processes, phagocytosis of apoptotic cells, epithelial cell polarization, neurons adhesion, migration and differentiation, and growth-factor induced formation of membrane ruffles
Q05144	Rac2	Rac Family Small GTPase 2	Cytosol, mitochondrion, extracellular, plasma membrane	GTPase activity, regulate cellular responses, such as secretory processes, phagocytose of apoptotic cells and epithelial cell polarization
P60764	Rac3	Rac Family Small GTPase 3	Cytosol, cytoskeleton, plasma membrane	GTPase activity, regulate cellular responses, such as cell spreading and the formation of actin-based protusions including lamellipodia and membrane ruffles
Q9ESK9	Rb1cc1	RB1 Inducible Coiled-Coil 1	Lysosome, cytosol, nucleus	Autophagy, molecular adaptor activity
P26043	Rdx	Radixin	Cytoskeleton, plasma membrane	Binding of the barbed end of actin filaments to the plasma membrane
O89026	Robo1	Roundabout Guidance Receptor 1	Plasma membrane	Cellular migration, axon guidance receptor activity
Q9CPR4	Rpl17	Ribosomal Protein L17	Cytosol	Structural constituent of ribosome
O09167	Rpl21	Ribosomal Protein L21	Cytosol	Structural constituent of ribosome
P67984	Rpl22	Ribosomal Protein L22	Cytosol, nucleus	Structural constituent of ribosome
Q8BP67	Rpl24	Ribosomal Protein L24	Cytosol	Structural constituent of ribosome

Uniprot ID	Gene	Short name	Subcellular location	Gene ontology
P47915	Rpl29	Ribosomal Protein L29	Cytosol	Structural constituent of ribosome
P62900	Rpl31	Ribosomal Protein L31	Cytosol	Structural constituent of ribosome
Q9D1R9	Rpl34	Ribosomal Protein L34	Cytosol	Structural constituent of ribosome
P47964	Rpl36	Ribosomal Protein L36	Cytosol, nucleus	Structural constituent of ribosome
P61514	Rpl37a	Ribosomal Protein L37a	Cytosol, extracellular	Structural constituent of ribosome
P62892	Rpl39	Ribosomal Protein L39	Cytosol, extracellular	Structural constituent of ribosome
P47962	Rpl5	Ribosomal Protein L5	Cytosol, nucleus	Structural constituent of ribosome, Mrna 3'-UTR binding, 5S rRNA binding
P62245	Rps15a	Ribosomal Protein S15a	Cytosol	Structural constituent of ribosome
P62270	Rps18	Ribosomal Protein S18	Cytosol, nucleus	Structural constituent of ribosome
P62849	Rps24	Ribosomal Protein S24	Cytosol	Structural constituent of ribosome
Q6ZWU9	Rps27	Ribosomal Protein S27	Cytosol, nucleus	Structural constituent of ribosome
P62983	Rps27a	Ribosomal Protein S27a	Cytosol, extracellular	Structural constituent of ribosome, protein tag, ubiquitin protein ligase binding
Q6ZWY3	Rps27l	Ribosomal Protein S27 Like	Nucleus	Structural constituent of ribosome, translation activator activity, apoptotic process
P14206	Rpsa	Ribosomal Protein SA	Cytosol, nucleus, plasma membrane	Structural constituent of ribosome, laminin receptor activity, cell fate determination and tissue morphogenesis
Q8BVY0	Rsl1d1	Ribosomal L1 Domain Containing 1	Nucleus	Cellular senescence, pro-apoctotic regulation, cadherin binding, mRNA 3' and 5' UTR binding
Q8BPR0	Rusc2	RUN And SH3 Domain Containing 2	Cytosol, extracellular	Vesicular trafficking, small GTPase binding
Q9D708	S100a16	S100 Calcium Binding Protein A16	Cytosol, nucleus, extracellular	Calcium-binding protein, protein homodimerizacion activity
P14069	S100a6	S100 Calcium Binding Protein A6	Cytosol, nucleus, plasma membrane	Cellular calcium signaling, reorganization of the actin cytoskeleton, cell motility.
Q9CY58	Serbp1	SERPINE1 MRNA Binding Protein 1	Cytosol, nucleus	Regulation of mRNA stability, translation repressor activity, SUMO binding
P19324	Serpinh1	Serpin Family H Member 1	Endoplasmic reticulum	Binds specifically to collagen, serine-type endopeptidase inhibitor activity
Q9EQU5	Set	SET Nuclear Proto-Oncogene	Cytosol, endoplasmic reticuñum, nucleus	Involved in apoptosis, transcription, nucleosome assembly and histone chaperoning.
Q99NB9	Sf3b1	Splicing Factor 3b Subunit 1	Nucleus	Involved in pre-mRNA splicing

Uniprot ID	Gene	Short name	Subcellular location	Gene ontology
Q921M3	Sf3b2	Splicing Factor 3b Subunit 2	Nucleus	Involved in pre-mRNA splicing
Q921M3	Sf3b3	Splicing Factor 3b Subunit 3	Nucleus	Involved in pre-mRNA splicing
Q91VW3	Sh3bgrl3	SH3 Domain Binding Glutamate Rich Protein Like 3	Nucleus	Modulator of glutaredoxin biological activity, cytoskeleton organization
Q8VEM8	Slc25a3	Solute Carrier Family 25 Member 3	Mitochondrion	Transport of phosphate groups from the cytosol to the mitochondrial matrix, regulation of the mitochondrial permeability transition pore (mPTP)
P51881	Slc25a5	Solute Carrier Family 25 Member 5	Mitochondrion, cytoskeleton	ATP:ADP antiporter activity, proton transmembrane transporter activity, ubiquitin protein ligase binding
P70441	Slc9a3r1	NHERF Family PDZ Scaffold Protein 1	Cytoskeleton, plasma membrane	Scaffold protein, actin regulation, cAMP-mediated phosphorylation, enhance Wnt signaling, sperm capacitation
Q9DB10	Smdt1	Single-Pass Membrane Protein With Aspartate Rich Tail 1	Nucleus, mitochondrion	Regulatory subunit of the mitochondrial calcium uniporter complex
P62317	Snrpd2	Small Nuclear Ribonucleoprotein D2 Polypeptide	Cytosol, nucleus	Pre-mRNA splicing
P62307	Snrpf	Small Nuclear Ribonucleoprotein Polypeptide F	Cytosol, nucleus	Pre-mRNA splicing
O70492	Snx3	Sorting Nexin 3	Cytosol, endosome	Multivesicular body formation, regulation of phagocytosis in dendritic cells, iron homeostasis, protein phosphatase binding
O70554	Sprr2b	Small Proline Rich Protein 2B	Cytosol, plasma membrane	Cross-linked envelope protein of keratinocytes
O70555	Sprr2d	Small Proline Rich Protein 2D	Cytosol, plasma membrane	Cross-linked envelope protein of keratinocytes
O70558	Sprr2g	Small Proline Rich Protein 2G	Cytosol, plasma membrane	Cross-linked envelope protein of keratinocytes
O70559	Sprr2h	Small proline-rich protein 2H	Cytosol, plasma membrane	Cross-linked envelope protein of keratinocytes
O70562	Sprr2k	Small proline-rich protein 2K	Cytosol, plasma membrane	Cross-linked envelope protein of keratinocytes
Q8BMA6	Srp68	Signal Recognition Particle 68	Cytosol, nucleus	Mediates the cotranslational targeting of secretory and membrane proteins to the endoplasmic reticulum (ER)
F8VQC1	Srp72	Signal Recognition Particle 72	Cytosol, endoplasmic reticulum	Mediates the cotranslational targeting of secretory and membrane proteins to the endoplasmic reticulum (ER)
Q9R0U0	Srsf10	Serine And Arginine Rich Splicing Factor 10	Nucleus	repressor of pre-mRNA splicing

Uniprot ID	Gene	Short name	Subcellular location	Gene ontology
Q8C8K3	Srsf12	Serine And Arginine Rich Splicing Factor 12	Nucleus	Antagonize SR proteins in pre-mRNA splicing regulation
Q8VE97	Srsf4	Serine And Arginine Rich Splicing Factor 4	Nucleus	Alternative splice site selection during pre-mRNA splicing
O35326	Srsf5	Serine And Arginine Rich Splicing Factor 5	Nucleus	Modulate the selection of alternative splice sites
Q3TWW8	Srsf6	Serine And Arginine Rich Splicing Factor 6	Nucleus	Modulate the selection of alternative splice sites
P54227	Stmn1	Stathmin 1	Cytosol, cytoskeleton	Regulation of the microtubule (MT) filament system by destabilizing microtubules
P11031	Sub1	SUB1 Regulator Of Transcription	Nucleus	RNA polymerase II cis-regulatory region sequence-specific DNA binding, DNA helicase activity, double-stranded DNA binding
P70279	Surf6	Surfeit 6	Nucleus	DNA and RNA binding
P11983	Tcp1	T-Complex 1	Cytosol, cytoskeleton	Component of the chaperonin-containing T-complex (TRiC), fold of actin and tubulin
Q91ZK0	Tfap2d	Transcription Factor AP-2 Delta	Nucleus	Regulate transcription of selected genes
P26039	Tln1	Talin 1	Cytosol	Phosphatidylserine binding, structural constituent of cytoskeleton
Q71LX4	Tln2	Talin 2	Cytoskeleton	Structural constituent of cytoskeleton, cell adhesion
Q9Z321	Top3b	DNA Topoisomerase III Beta	Nucleus	DNA and RNA binding, DNA topoisomerase activity
P58771	Tpm1	Tropomyosin 1	Cytoskeleton	Binds to actin filaments in muscle and non-muscle cells, calcium dependent regulation, smooth muscle contraction, stabilizization of cytoskeleton actin filaments
P58774	Tpm2	Tropomyosin 2	Cytosol	Binds to actin filaments in muscle and non-muscle cells, calcium dependent regulation, smooth muscle contraction, stabilizization of cytoskeleton actin filaments
P21107	Tpm3	Tropomyosin 3	Cytosol, cytoskeleton, extracellular	Binds to actin filaments in muscle and non-muscle cells, calcium dependent regulation, smooth muscle contraction, stabilizization of cytoskeleton actin filaments
Q6PFY8	Trim45	Tripartite Motif Containing 45	Cytosol, nucleus	transcriptional repressor in mitogen-activated protein kinase signaling pathway, ubiquitin protein ligase activity
Q792Z1	Try10	Trypsin 10	Extracellular	Proteolysis
P68369	Tuba1a	Tubulin alpha-1A chain	Cytosol, cytoskeleton	Adult behavior, cellular response to calcium ion, centrosome cycle
P05213	Tuba1b	Tubulin alpha-1B chain	Cytosol, cytoskeleton	Cellular response to IL-4, microtubule cytoskeleton organization, mitotic cell cycle

Uniprot ID	Gene	Short name	Subcellular location	Gene ontology
P68373	Tuba1c	Tubulin alpha-1C chain	Nucleus, cytoskeleton	Microtubule cytoskeleton organization, mitotic cell cycle
P05214	Tuba3a	Tubulin alpha-3 chain	Cytoskeleton	Male-germ-line stem cell population maintenance, mictotubule cytoskeleton organization, mitotic cell cycle
P68368	Tuba4a	Tubulin alpha-4A chain	Cytosol, cytoskeleton	Microtubule cytoskeleton organization, mitotic cell cycle
Q9JJZ2	Tuba8	Tubulin alpha-8 chain	Cytoskeleton	Microtubule cytoskeleton organization, mitotic cell cycle, spermatid development, spermatogenesis
P26369	U2af2	Splicing factor U2AF 65 kDa subunit	Nucleus	mRNA splicing, negative regulation of protein ubiquitination
P62984	Uba52	Ubiquitin A-52 Residue Ribosomal Protein Fusion Product 1	Cytosol	Cytoplasmic translation, modification-dependent protein catabolic process, protein ubiquitination
P0CG49	Ubb	Polyubiquitin-B	Cytosol, nucleus, mitochondrion	Adipose tissue development, energy homeostasis, female gonad development
P0CG50	Ubc	Polyubiquitin-C	Cytosol, nucleus, mitochondrion	Modification-dependent protein catabolic process
Q5FWH2	Unkl	Putative E3 ubiquitin-protein ligase UNKL	Nucleus	Protein ubiquitination
Q64727	Vcl	Vinculin	Cytosol, extracellular, plasma membrane	Adherens junction assembly, apical junction assembly, axon extension, cell adhesion
E9Q0K5	Vmn2r63	Vomeronasal 2	-	-
Q920Q4	Vps16	Vacuolar protein sorting-associated protein 16 homolog	Lysosome, endosome	Autophagosome maturation, endosomal transport, intracellular protein transport
Q9CQV8	Ywhab	14-3-3 protein beta/alpha	Cytosol	Cytoplasmic sequestering of protein, negative regulation of DNA-templated transcription, positive regulation of catalytic activity
P62259	Ywhae	14-3-3 protein epsilon	Cytosol, nucleus	Cellular response to heat, cerebral cortex development, MAPK cascade
P61982	Ywhag	14-3-3 protein gamma	Cytosol	Cellular response to insulin stimulus, protein targeting, regulation of neuron differentiation
P68510	Ywhah	14-3-3 protein eta	Cytosol	Cytoskeleton organization, glucocorticoid catabolic process, intracellular protein transport
Q9ESX4	Zcchc17	Zinc finger CCHC domain-containing protein 17	Nucleus	RNA stabilization
B2RR24	Zfp236	Zinc finger protein 236	Nucleus	Regulation of transcription by RNA polymerase II
Q8C6P8	Zfp57	Zinc finger protein 57	Nucleus	DNA methylation involved in embryo development, genomic imprinting, negative regulation of gene expression via CpG island methylation, negative regulation of transcription by RNA pol II
